# The Panacea of Modern Gynecology

**Published:** 1886-06-20

**Authors:** 


**The Panacea of Modern Gynecology.**—Winckel, in his *Lehrbuch der Frauenkrankheiten*, which has just been published, speaks of laparotomy as the panacea of modern gynecology. Such conservatism is not in correspondence with the spirit of the times when so many speak of "Batty," or of a "Tait," as a frequent event, and regard extirpation of the ovaries, or of the tubes, or of both, as an operation the indications for which are often presented and are unequivocal. Possibly, however, the voice of conservatism ought to be heard in this, as well as in some other departments of operative gynecology. If the history of some at least of those whose sound ovaries have been removed where given months, or a year or two after the extraction, it would be found that no good had resulted. Winckle regards the time as not far distant when the extirpation of the healthy ovaries for the cure of dysmenorrhœa, ovaralgia, epilepsie, hysteria, and mental disorders will be considered in the same light as clitoridectomy, except that the latter was much more innocent and harmless.—*Medical News.*

